# Inbred mouse strains C57BL/6J and DBA/2J vary in sensitivity to a subset of bitter stimuli

**DOI:** 10.1186/1471-2156-6-36

**Published:** 2005-06-20

**Authors:** John D Boughter, Sandeep Raghow, Theodore M Nelson, Steven D Munger

**Affiliations:** 1 Anatomy and Neurobiology, University of Tennessee Health Science Center, Memphis, TN 38163 USA; 2 Anatomy and Neurobiology, University of Maryland School of Medicine, Baltimore, MD 21201, USA; 3 Program in Neuroscience, University of Maryland School of Medicine, Baltimore, MD 21201, USA

## Abstract

**Background:**

Common inbred mouse strains are genotypically diverse, but it is still poorly understood how this diversity relates to specific differences in behavior. To identify quantitative trait genes that influence taste behavior differences, it is critical to utilize assays that exclusively measure the contribution of orosensory cues. With a few exceptions, previous characterizations of behavioral taste sensitivity in inbred mouse strains have generally measured consumption, which can be confounded by post-ingestive effects. Here, we used a taste-salient brief-access procedure to measure taste sensitivity to eight stimuli characterized as bitter or aversive in C57BL/6J (B6) and DBA/2J (D2) mice.

**Results:**

B6 mice were more sensitive than D2 mice to a subset of bitter stimuli, including quinine hydrochloride (QHCl), 6-n-propylthiouracil (PROP), and MgCl_2_. D2 mice were more sensitive than B6 mice to the bitter stimulus raffinose undecaacetate (RUA). These strains did not differ in sensitivity to cycloheximide (CYX), denatonium benzoate (DB), KCl or HCl.

**Conclusion:**

B6-D2 taste sensitivity differences indicate that differences in consumption of QHCl, PROP, MgCl_2 _and RUA are based on immediate orosensory cues, not post-ingestive effects. The absence of a strain difference for CYX suggests that polymorphisms in a T2R-type taste receptor shown to be differentially sensitive to CYX *in vitro *are unlikely to differentially contribute to the CYX behavioral response *in vivo*. The results of these studies point to the utility of these common mouse strains and their associated resources for investigation into the genetic mechanisms of taste.

## Background

The majority of heritable traits in humans and other species are complex in nature, determined by interactions among multiple genes and environmental factors. Mouse genetic models have been critical in identifying genes that determine complex behavioral traits (e.g., [1-3]). The standard inbred strains C57BL/6J (B6) and DBA/2J (D2) have played a key role in mouse genetics, and they are among the strains included in the public and private genome sequencing projects. BXD/Ty (BXD) recombinant inbred (RI) mice, created from B6 and D2 progenitors, have been used to identify and map quantitative trait loci (QTLs) that influence diverse phenotypes such as addictive behavior (e.g., [4-6]), lifespan [7], central nervous system anatomy [8-10], and solution consumption [11-13]. Recently, the BXD set has been expanded to about 80 strains, which makes it the largest mouse RI mapping panel and a useful resource for QTL analysis [14].

The characterization of behavioral taste sensitivity in B6 and D2 mice would facilitate the use of BXD mice in mapping QTLs for taste sensitivity (56). B6 mice have been the most common inbred strain used in gustatory research, and as such have been characterized in terms of one- and two-bottle intake, brief-access taste sensitivity, operant taste detection tasks, taste discrimination, gustatory nerve physiology, taste receptor cell physiology, and taste cell-specific gene expression (e.g., [15-23]. D2 mice have not been as thoroughly characterized (e.g., [23-25]. However, it has long been appreciated that significant differences between B6 and D2 mice exist for consumption of sweet- and bitter-tasting stimuli, ethanol, and sodium chloride [26-29].

For stimuli characterized by humans as possessing a bitter taste, B6 and D2 mice vary in level of responsiveness when queried with intake tests: concentration-dependent strain differences have been measured for the typical bitter stimulus quinine hydrochloride (QHCl; [22,28]), acetylated sugars such as raffinose undecaacetate (RUA; [11,30]), and copper glycinate [13]. Such two-bottle intake procedures have been manageable for testing the large numbers of mice required for quantitative analysis. However, it is questionable whether these tests provide valid indicators of an animal's ability to recognize or discriminate a substance based on gustatory cues. Bitter stimuli comprise an exceptionally diverse set of chemical compounds that vary greatly in toxicity (e.g., [31,32]). Our recent study [33] demonstrates that post-ingestive effects related to bitter stimulus toxicity directly influence results from two-bottle tests and that these effects are considerably minimized in brief-access tests. Here we describe the use of a taste-salient brief-access procedure (e.g., [16,33,34] to characterize taste sensitivity in B6 and D2 mice to eight stimuli: six which are perceived by humans as predominantly bitter-tasting (QHCl, 6-n-propylthiouracil (PROP), MgCl_2_, RUA, denatonium benzoate (DB), and cycloheximide (CYX)), one with a complex salt/bitter taste (KCl), and an acid (HCl). These studies demonstrate that B6 and D2 mice differ in taste sensitivity to some, but not all, bitter or aversive stimuli, and suggest that they will be a useful resource for characterizing the genetic basis of bitter taste.

## Results

### Response to bitter and acid stimuli

**Figure 1:**
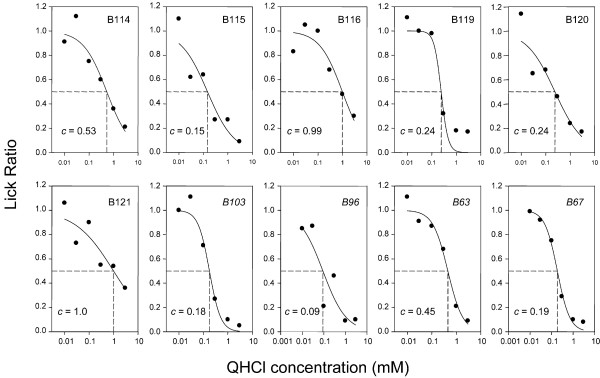
Concentration-response functions quinine in 10 individual B6 mice. Data points for each mouse represent average ratios across two days of testing; these means were fitted with two-parameter logistic functions and the concentration evoking half-maximal avoidance, *c*, was estimated. Italicized mice (*B103, B96, B93, B67*) were given QHCl as the last of three stimuli, as opposed to the others, which received QHCl as the first stimulus.

**Figure 2:**
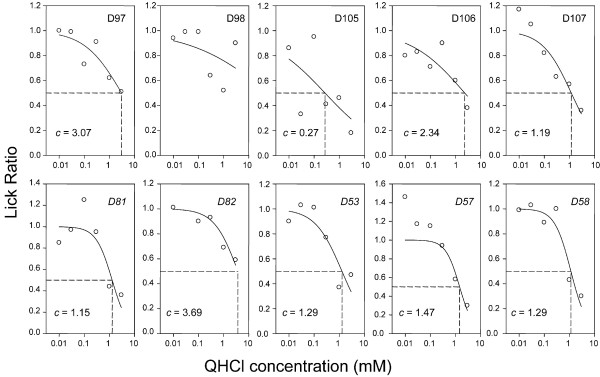
Concentration-response functions for QHCl in 10 individual D2 mice. Data points for each mouse represent average ratios across two days of testing; these means were fitted with two-parameter logistic functions and the concentration evoking half-maximal avoidance, *c*, was estimated. For one mouse (D98) this parameter could not accurately estimated, although this mouse had a mean lick ratio of 0.52 for 1 mM QHCl. Italicized mice (*D81, D82, D53, D57, D58*) were given QHCl as the last of three stimuli, as opposed to the others which received QHCl as the first stimulus.

Thirty B6 and 30 D2 mice were tested with six concentrations each of three different stimuli, such that a total of ~ 10 mice of each strain were tested for each of eight stimuli (Table 1; QHCl, PROP, MgCl_2_, RUA, DB, CYX, KCl, and HCl). Concentration-response functions were created for individual mice (for all compounds) and were fitted with two-parameter logistic functions, so that the concentration evoking half-maximal avoidance (*c*) could be determined. Examples of such individual functions for QHCl in B6 and D2 mice are shown in Figures 1 and 2, respectively. All B6 mice displayed concentration-dependent avoidance of QHCl (Fig. 1). Estimated half-maximal avoidance (*c*) ranged from 0.09 to 1.0 mM QHCl among individual mice, but there was not a significant difference between the six mice tested with QHCl as the first stimulus versus the four that were tested with QHCl as the third stimulus. Estimated half-maximal avoidance of QHCl among individual D2 mice ranged from 0.27 to 3.07 mM. For all but one D2 mouse (D105; Fig. 2), *c *> 1.15 mM. Some of the D2 mice such as D98, D97 and D82 showed relatively little avoidance of the higher concentrations of QHCl. As was the case for B6 mice, *c *did not vary significantly among individual mice as a function of whether the QHCL was presented as the first or last stimulus in the test series (D98 was not included in this comparison, because *c *could not be accurately estimated. However, all B6 and D2 mice were used for repeated measures comparisons; see below). The average half-maximal avoidance was 0.41 mM for B6 mice and 1.75 mM for D2 mice [*t*(17) = 4.33; *p *< 0.001]. Notably, comparisons of *c *within strain for all eight stimuli did not reveal significant effects of test group (i.e. whether the stimulus was presented first, second or third in series; see Table 1) with a single exception, noted below. Data collected for each compound were therefore combined for analysis of potential strain and gender effects.

**Table 1:** Test compounds and test series for 30 B6 and 30 D2 mice. Each mouse was tested with three compounds (2 consecutive test sessions per compound) over a two-week period. Stimuli were KCl, cycloheximide (CYX), raffinose undecaacetate (RUA), 6-n-propylthiouracil, quinine hydrochloride (QHCl), denatonium benzoate (DB), and HCl. A total of 10 mice were tested from each strain (5 males, 5 females) for KCl, CYX, PROP, MgCl_2_and QHCl. A total of 10 mice from each strain (5 males, 5 females for B6; 7 females, 3 males for D2) were tested with DB and HCl. A total of 11 B6 mice (6 females, 5 males) and 9 D2 mice (5 females, 4 males) were tested with RUA.

		Number of Mice Tested	
Squad	Compounds	B6	D2

			
A	KCl, CYX^1^	5	5
B	CYX, RUA, KCl	5	5
C	PROP, MgCl2, QHCl	2	3
D	MgCl2, PROP, QHCl	2	2
E	DB, HCl^2^	4	6
F	QHCl, MgCl2, PROP	6	5
G	HCl, DB, RUA	6	4
			
	Total Mice	30	30

^1,2^In squads A and E additional data on a third aversive stimulus was collected but was treated as pilot data and not included as part of the current experiment.

**Figure 3:**
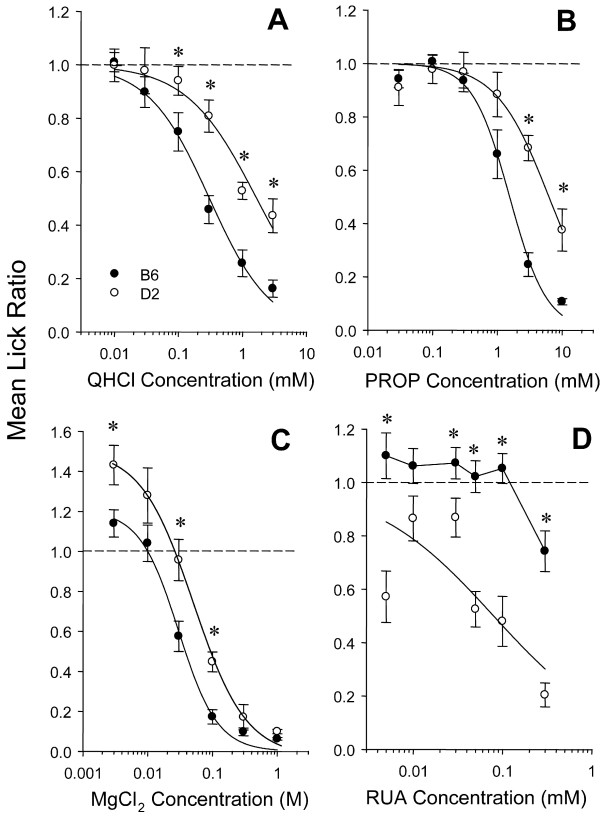
Lick ratios (mean ± SE) for B6 and D2 mice to concentration series of QHCl (A), PROP (B), MgCl_2 _(C), and RUA (D). The dotted lines on each graph represent a ratio score of 1.0, which indicates a lick rate equal to that of water. Asterisks identify significant strain effects at particular concentrations, as indicated by planned comparisons (*p *< 0.01). Lick ratios for each strain generally decreased with increasing concentration; B6 made fewer licks than D2 mice to high concentrations of QHCl (A) and PROP (B), and to both low and concentrations of MgCl_2 _(C). D2 mice made fewer licks than B6 mice at almost all concentrations of RUA (D).

Strain differences in taste sensitivity were found for four of eight compounds: QHCl, PROP, MgCl_2_, and RUA (Figure 3). For QHCl, D2 mice had significantly higher lick ratios than B6 mice across most of the concentration range, indicating decreased aversion. A strain x gender x concentration ANOVA revealed a main effect of strain [F(1,16) = 16.64, p < 0.001] but not gender. There was a significant strain x concentration interaction [F(5,80) = 3.74, p < 0.01]. The strain x gender, or strain x gender x concentration interactions were not significant for QHCl (or for any of the 7 other stimuli). Planned comparisons (Least Squares means) between strain at each concentration revealed that D2 mice were significantly less sensitive (p < 0.01) to 0.3 – 3 mM QHCl.

Similarly, D2 mice were less sensitive to PROP: a strain x gender x concentration ANOVA revealed a main effect of strain [F(1,16) = 10.5, p < 0.01] but not gender. There was a significant strain x concentration interaction [F(5,80) = 7.01, p < 0.0001]. Planned comparisons indicated D2 mice possessed higher lick ratios at 3 and 10 mM. The mean half-maximal avoidance was 1.46 mM for B6 mice and for 4.97 mM for D2 mice [*t*(17) = 4.05; *p *< 0.001].

For MgCl_2_, there was a main effect of strain [F(1,16) = 12.6, p < 0.01] but not gender. Planned comparisons indicated D2 mice possessed significantly higher lick ratios at 0.003, 0.1 and 0.3 mM. A modest curve shift across the entire concentration range was not quite significant: mean half-maximal avoidance was 0.02 M for B6 mice and 0.05 M for D2 mice [*t*(17) = 2.34; *p *= 0.03].

For RUA, the direction of the strain difference was reversed. Notably, B6 mice did not display strong aversion to any concentration, whereas D2 mice avoided RUA in a concentration-dependent manner. There was a main effect of strain [F(1,16) = 39.56, p < 0.0001] but not gender. A significant interaction was found for concentration x strain [F(5,80) = 3.84, p < 0.01]. Planned comparisons indicated D2 possessed greater avoidance than B6 mice at 0.005, and at 0.03–0.3 mM. D2 mice actually possessed a greater level of avoidance to the lowest concentration of RUA (0.005 mM) relative to the next two higher concentrations (0.01 and 0.03). This tendency was evident in eight of nine individual D2 mice (data not shown), although it is not clear why such an effect was found. The mean curve shift between strains was not examined for RUA due to the lack of concentration-dependent avoidance in B6 mice, and the resulting inability to estimate the *c-*parameter.

**Figure 4:**
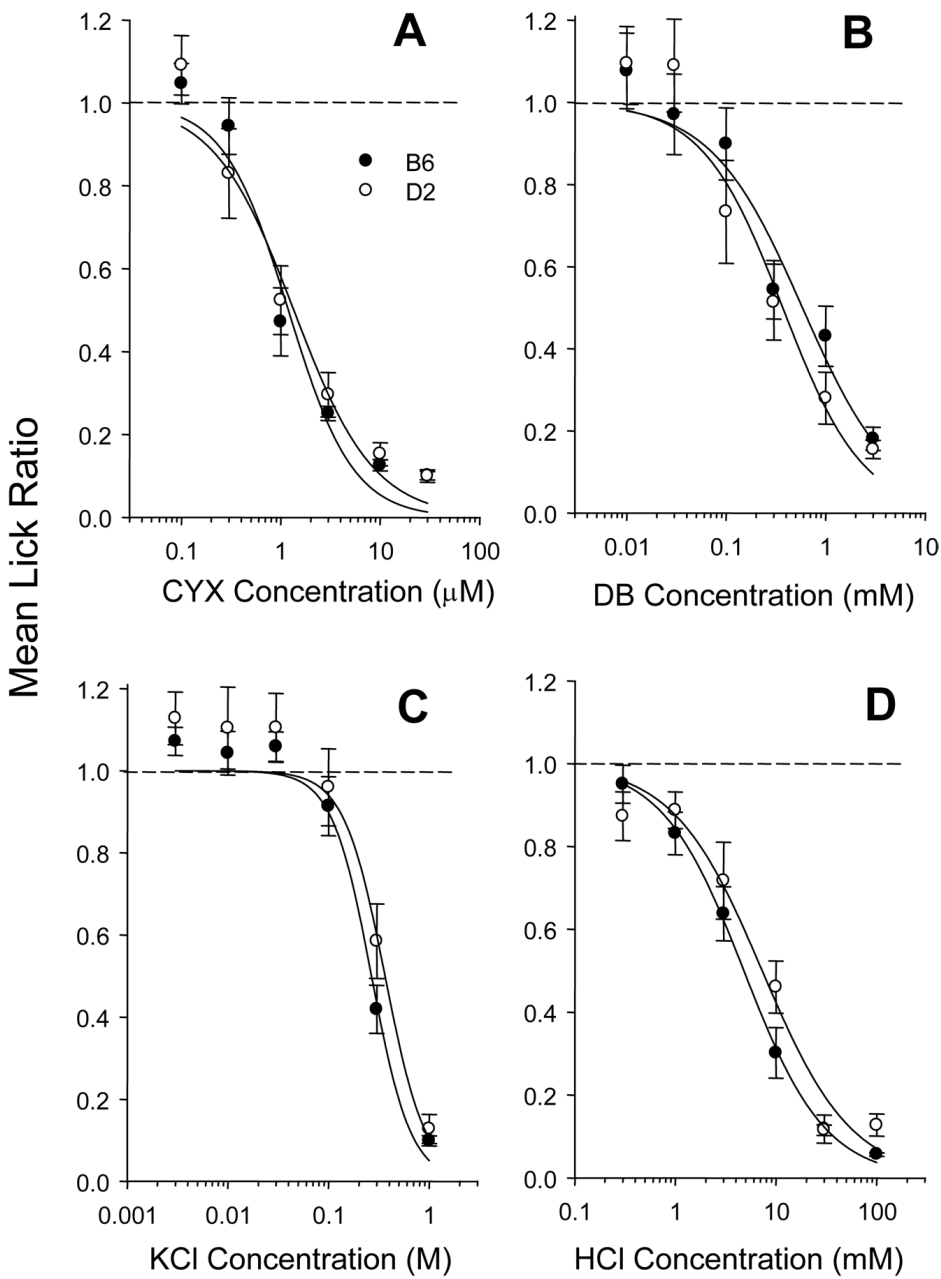
Lick ratios (mean ± SE) for B6 and D2 mice to concentration series of CYX (A), DB (B), KCl (C), and HCl (D). The dotted lines on each graph represent a ratio score of 1.0, which indicates a lick rate equal to that of water. Lick ratios for each strain decreased with increasing concentration. The strains did not differ significantly for any of these compounds.

We did not detect strain or gender differences in sensitivity to CYX, DB, KCl, or HCl (Figure 4). Both strains avoided higher concentrations of each of these stimuli in a concentration-dependent fashion. An effect on half-maximal avoidance based on test series was found for CYX: D2 mice that received CYX as the first stimulus tended to have a lower *c *value (mean = 0.53 μM) than those that received it as the second stimulus (mean = 2.66 μM; *p *< 0.01). A similar effect was found in the B6 mice, although not quite significant (*p *= 0.03). Testing each of these subgroups for significance with ANOVA (with n = 5 / strain) showed that B6 and D2 still did not differ in level of aversion (p > 0.08; however, small sample sizes in this comparison should be noted). Between conditions, lick ratios for both strains tended to differ modestly at 0.3 and 1 μM, but not at the higher concentrations (3–100 μM). The cause of the within-strain curve shifts is unclear, although B6 and D2 mice did not differ significantly in sensitivity to this stimulus.

### Baseline licking, performance and latency

Taste data were reported as lick ratios in order to standardize for possible strain differences in water lick rate [16,33]. We compared these "baseline" rates of water licking between B6 and D2 mice collapsed across all days of stimulus testing. B6 mice licked water at an average rate of 33.81 licks / 5 s whereas D2 licked at an average rate of 35.76 licks / 5 s; this difference was not significant [F(1,58) = 2.68; *p *> 0.1]. In addition to lick rate, we were also interested in examining other aspects of behavior in the task that are thought to be non-gustatory in origin. *Performance *was reported as percent trials completed per test session, per mouse. Table 2 lists mean performance rates for each strain, by gender, for each of three consecutive test sessions. In general, B6 mice of either gender tended to complete a slightly larger percentage of trials than did D2 mice [F(1,52) = 17.10, *p *< 0.01]. In order to account for possible effects of each unique stimulus on performance, we also examined this performance for each of the eight stimuli: There was still a significant main effect of strain [F(1,147) = 20.9; p < 0.001], although the effect for stimulus was not significant.

**Table 2:** Mean % trials completed by stimulus order. A trial was considered to be complete as long as a mouse took a single lick. After 120 s had elapsed without a lick, a given trial was considered over, and the next trial began. B6 mice completed significantly more trials (out of a possible 24 per session) than did D2 mice across all three stimuli with which those mice were tested.

		*n*	Stim 1	Stim 2	Stim 3
Strain	Gender				

					
B6	Female	15	96.9	94.6	96.0
	Male	15	93.1	91.2	90.6
					
D2	Female	17	96.6	93.6	97.2
	Male	13	93.4	88.6	94.6

**Figure 5:**
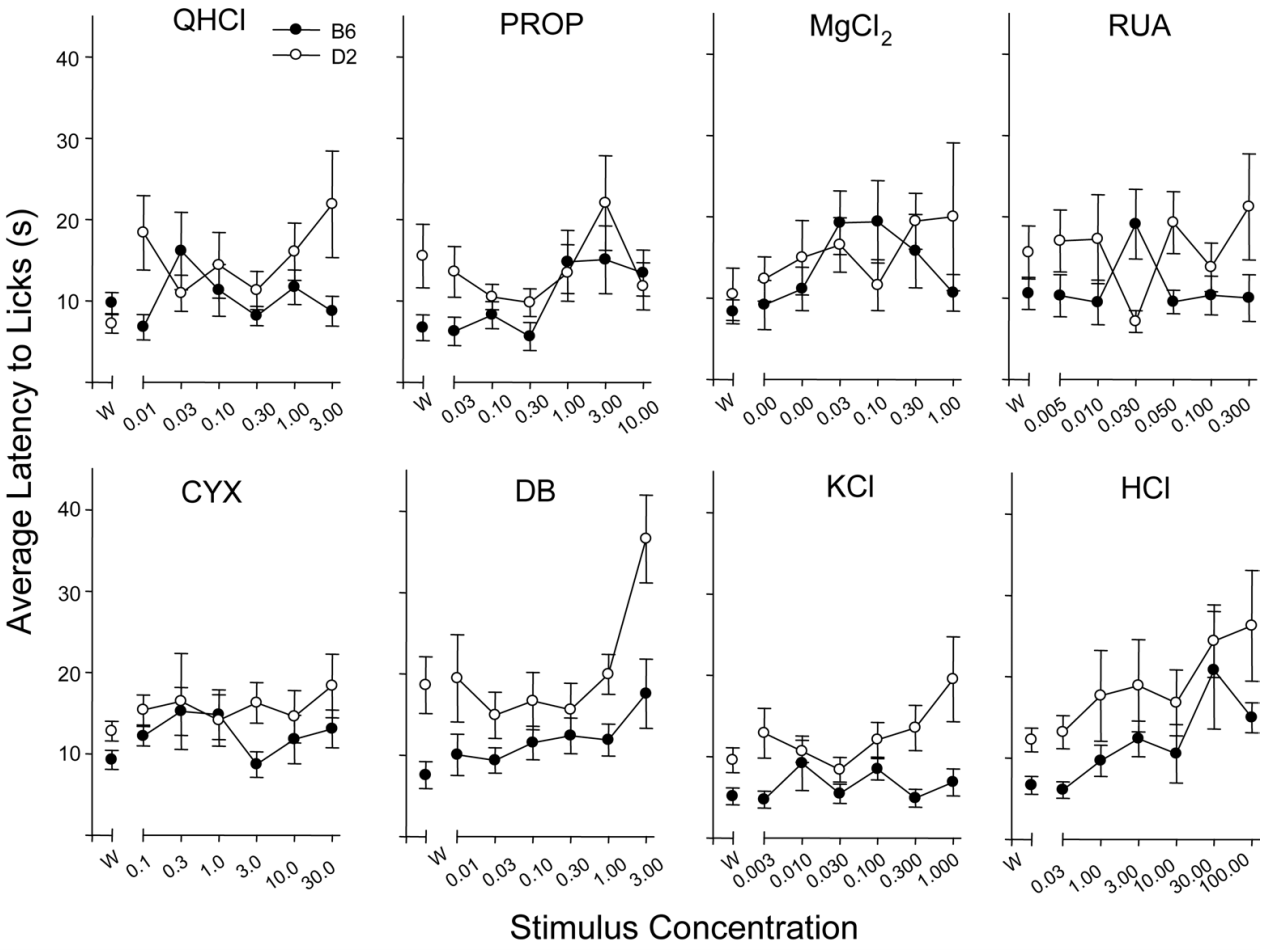
Strain comparison of latency to initiate taste trials of all eight stimuli. W = water trials. Latencies are means (± SE) of the median latencies for individual mice. A significant strain difference was found only for KCl. Effects of concentration were found for DB and HCl.

We asked if olfactory cues might contribute to avoidance in the brief-access test. We measured the latency to initiate trials for each compound, because concentration-dependent changes in latency have been suggested previously to indicate olfactory contribution [35]. Latencies for all stimuli are shown in Figure 5. A significant main effect of strain was found for only KCl [F(1,160) = 9.96, p < 0.01], whereas effects of concentration were significant for DB and HCl [F(6,96) > 3.4, p < 0.01]. Only a single significant interaction was detected: Concentration x strain for RUA [F(6,96 = 3.15; p < 0.01]. In general, greater latency was often observed for the highest concentration of a given stimulus. Latency to lick may increase as a function of olfactory cues, but it is important to consider that other strain differences such as overall activity levels may also affect this measure. Overall, reliable effects on latency to lick bitter stimuli in mice have not been reported [33,34,36].

## Discussion

Previous studies comparing the consumption of bitter stimuli in two or more standard inbred strains of mice have fostered genetic and molecular approaches towards identifying taste transduction mechanisms (e.g., [11,12,28,37-39]). Given the potential for which post-ingestive factors affect levels of intake of particular bitter stimuli [33], we were eager to assess taste sensitivity in the commonly used inbred strains B6 and D2 with a taste-salient brief-access assay. We demonstrated significant concentration-dependent differences in taste sensitivity to a subset of aversive compounds, including the bitter stimuli QHCl, PROP, MgCl_2_, and RUA. Strain differences were not found for CYX, KCl, DB, or HCl. These strains did not differ in baseline levels of water licking. Additionally, we did not find effects of gender on taste sensitivity.

The B6-D2 taste sensitivity differences demonstrated for QHCl and RUA indicate that differences in consumption of these stimuli between these strains [22,28] are based on immediate orosensory cues. Our results are also consistent with a potential relationship between quinine and PROP aversion [37]. D2 mice displayed significantly less aversion than B6 mice to both QHCl and PROP; the strain differences were similar in magnitude (Fig. 4). The relationship between quinine and PROP taste sensitivity is surprising when one considers that, in contrast to the situation in humans, taste sensitivity to PROP in mice is not correlated with its structural analogue phenylthiocarbamide (PTC) [33].

Linkage studies have postulated a major locus controlling quinine intake, *qui*, closely linked to *Prp2 *and *Prh1 *(which encode two proline-rich salivary proteins) and the microsatellite marker D6Mit13 on distal mouse chromosome 6 [21,37,40]. Harder and Whitney [37] further showed, using BXH RI mice (bred from progenitor strains B6 and C3HeB/HeJ), that PROP intake was also under Chr 6 control. The linkage of putative bitter taste receptor genes (*Tas2rs*) to these loci predicts that polymorphisms in particular *Tas2rs *underlie strain differences to these stimuli. However, the ligand specificity of these receptors is difficult to predict, given that only a handful of mouse or human bitter taste receptors have been functionally characterized, and that evidence for narrow or broad tuning is equivocal [41-43]. There is strong evidence for polygenic control of both QHCl and PROP intake based on quantitative genetic analyses [15,37,44,45], and *Tas2r-*independent mechanisms for QHCl taste have been suggested (e.g., [46,47]). A genetic analysis using the brief-access assay may help to determine whether non-*Tas2r *genes contribute to quinine sensitivity based on immediate sensory cues (56).

We also found that D2 mice were less sensitive for MgCl_2_, a compound not previously investigated in these strains. Interestingly, D2 mice licked the lowest concentration of MgCl_2 _(0.003 mM) at a rate greater than distilled water. We previously reported that C3HeB/FeJ inbred mice prefer 0.01 and 0.03 mM MgCl_2 _to water in a two-bottle intake test [33]. It is possible that water-deprived mice, as used in this study, will lick water at a maximal rate, making an appetitive response difficult to discern. However, Dotson and Spector [25] demonstrated that water-restricted mice of some strains will lick certain concentrations of appetitive stimuli (e.g., sucrose and glycine) at a higher rate than water. In our study, water licking rates during the MgCl_2 _test session did not significantly differ for those collected during testing with other stimuli. At higher concentrations (0.3–1.0 mM), MgCl_2 _was strongly avoided by both strains. This stimulus is perceived as having both a salty and bitter taste to humans [48]; in neural recordings with macaques it generally correlates with other bitters [49]. It is possible that at lower concentrations it is preferred over distilled water by some strains of mice for its salt taste component. MgCl_2 _is an intriguing candidate for which to determine possible *Tas2r *linkage; it evokes a strong anterior tongue-related neural response [50], which is a region that does not include strong T2R expression.

RUA is a non-toxic acetylated sugar; substantial variation in intake levels among inbred mouse strains have been shown for RUA as well another acetylated sugar, sucrose octaacetate (SOA) [11,51,52]. Sensitivity to SOA is determined by allelic variation at *Soa*, a locus linked to *Prp *and the *Tas2r *cluster (e.g., [38,53]). A separate locus, *Rua*, was proposed to control aversion to RUA 11. In view of the close chemical similarity of RUA and SOA, and given identical inbred strain distribution patterns (SDPs), a more parsimonious explanation is that *Soa *and *Rua *are identical loci [13,30]. In our experiment, RUA taste sensitivity provides an example where D2 mice display greater levels of avoidance than B6 mice, demonstrating the specific nature of the bitter avoidance response in these two strains.

B6 and D2 mice did not significantly differ in taste sensitivity to DB, KCl, HCl and CYX. The non-difference for CYX is especially interesting in that there are several coding-region polymorphisms in the *Tas2r105 *receptor (initially called T2R5) gene between these strains [41]; 56); this receptor is activated by CYX in diverse *in vitro *assays, and a shift in the concentration-response function indicates that the B6 isoform is less sensitive [41]. This finding has led to the erroneous conclusion that B6 mice "cannot detect cycloheximide" (e.g., [54]). In fact, our finding that D2 and B6 mice do not differ significantly in taste-based aversion of CYX (Fig. 4) supports the previous finding that these strains do not differ in consumption [12]; see Fig. 3B and 3C). Additionally, CYX evokes a peripheral (glossopharyngeal) nerve response in B6 mice comparable to that evoked by PROP [18]. It is likely that other T2Rs, or T2R-independent mechanisms, contribute to the behavioral response in B6 mice and mask any potential difference produced by the polymorphic receptor 55. Alternatively, it is possible that a subtle, yet significant, strain difference might be detected with the testing of a larger sample of mice, or with the use of a different procedure such as threshold detection (e.g., [17]).

Our results point to the utility of these common inbred strains and their associated genetic resources, such as the BXD strains, for investigation into the genetic mechanisms of taste. Although behavioral sensitivity to bitter-tasting stimuli has been linked to the *Tas2r *family of bitter taste receptor genes, both by positional or functional studies, compelling questions remain about the peripheral and central organization of bitter taste and the relative contributions of individual genes to specific taste sensitivities.

## Methods

### Mice

A total of 60 male and female mice (*Mus musculus*) from inbred strains were used in these experiments: 30 from each inbred strain (B6 and D2). Mice were tested at an average age of 3.5 months, and were age-matched between strains in each test group. Table 1 lists the number of mice tested in each experiment. Roughly equal numbers of B6 and D2 mice were tested with up to 3 different stimuli over a two-week period. Mice were obtained directly from the Jackson Laboratory (Bar Harbor, ME), or were derived (first generation) from mice obtained from Jackson Laboratory. Mice were housed in plastic home cages (28 × 17.5 × 13 cm) with stainless steel wire lids. Food (Teklad 8640 rodent diet) and water were available *ad lib*. Immediately prior to water deprivation, the mean weight of B6 mice was 20.1 g (females) and 27.4 g (males); the mean weight of D2 mice was 21.6 g (females) and 26.2 g (males).

### Apparatus

Mice were tested daily in the Davis MS-160 automated gustometer (DiLog Instruments, Inc., Tallahassee, FL). The test chamber consisted of a plastic rectangular cage (30 × 14.5 × 18 cm) with a wire mesh floor; an oval opening centered in the front wall allows access to water or taste solutions contained in leak-proof sipper tubes. Fluid access is restricted by a computer-operated shutter.

Trials began with the opening of the shutter and ended 5 s after the mouse made its first lick on the drinking spout (see Procedure). Licks were counted with a high-frequency AC contact circuit. Failure to initiate a lick within 120 s also ended a trial, although such "zero lick" trials were ignored in analyses of lick rate as the failure to initiate licking could not be ascribed to orosensory factors. In between trials, a platform upon which the stimulus bottles were mounted was advanced to a new position. The inter-trial interval was held constant at 10 s. The test session ended after the completion of 29 trials.

### Solutions

The taste stimuli used in these experiments were made from reagent-grade chemicals: 6-n-propylthiouracil (PROP), cycloheximide (CYX), raffinose undecaacetate (RUA), magnesium chloride (MgCl_2_), potassium chloride (KCl), quinine hydrochloride (QHCl), denatonium benzoate (DB) and hydrochloric acid (HCl) (Sigma Aldrich Corp.; St. Louis, MO). Multiple concentrations of each solution were made fresh daily using distilled water, and all taste stimuli were presented at room temperature (concentrations are listed in Table 1).

### Brief-access tests

Water-deprived mice were trained to lick water in the gustometer and subsequently tested with a six-concentration series of a taste stimulus. Mice were water-deprived for 24 h prior to the first day of training, and from that point on were restricted to water consumed during the training or testing session (approximately 1.5 ml per session). On the first training day, mice were placed in the test chamber and given access to distilled water for 20 min. Most mice took at least 100 licks from the drinking spout in this first session. On the second training day, access was restricted to 5 s trials. Water was delivered at random from one of four water tubes, and mice had the opportunity to initiate up to 16 trials. Testing with the first bitter stimulus occurred on days 3 and 4. Six concentrations of the stimulus plus water were delivered using a randomized block design. Twenty-four trials were divided into 3 blocks of 8; within each block, each concentration of the stimulus plus two water trials were presented in a random order. Although we have previously determined that most mice are sated after 24 5-s trials of both stimulus and water, a final block of five consecutive water trials were given in order to allow the thirstiest mice to rehydrate. Licking in these additional trials was not analyzed. In sum, each test session provided three possible data points per stimulus concentration, and six for water trials. The order of all trials was randomized anew for each mouse and the position of bottles on the gustometer was randomized each day. Finally, mice of both strains were tested in a random order each day.

After testing on day 4, mice received *ad lib *water in their home cages for 48 hours (over the weekend), followed by a second 24-h water deprivation prior to a single training session and consecutive two-day tests with two additional stimuli, conducted as described above. We tested 7 "squads" of mice this way, until testing of 10 mice from either strain for each of eight stimuli was completed (see Table 1).

### Data analysis of behavioral tests

The number of licks for each stimulus trial (each concentration being presented twice per mouse per session), plus water test trials, were averaged across the two test days for each individual mouse. These data were then reported as lick ratios (LR: average number of licks to stimulus / average number of licks during water test trial) in order to standardize for possible strain differences in water lick rate. Lick ratios thus range from a hypothetical zero (complete avoidance) to 1.0 (or greater). A ratio equal to zero was not possible because zero lick trials were not included in this analysis. Concentration-response functions were fit with a two-parameter logistic function:



Where *x *is the concentration of stimulus, *c *is the concentration evoking half-maximal avoidance (i.e. lick ratio = 0.5) and *b *is the slope. Fitting such curves provides a single parameter (*c*) that is sensitive to shifts in the concentration-response function, as potentially resulting from strain differences. For lick ratios in response to MgCl_2_, a three-parameter function was used; the additional parameter *a *was used to calculate an asymptotic maximum > 1.0, since lick ratios to the lowest concentration (0.003 M) of this tended to be greater than ~1.0, especially in D2 mice (see Figure 1). For group comparisons, *c *values were log transformed; strain values presented are therefore geometric means.

All relevant variables were analyzed using a general linear model: repeated measures (concentration) with between-subjects factors (strain, gender) and planned comparisons (LSD) at single concentrations based on the expectation of strain differences (Statistica software, StatSoft, inc., Tulsa, Oklahoma). Latency data (median latency) was log transformed for ANOVA. The statistical rejection criterion (α) for all tests was set *a priori *at the 0.01 level for main effects.

## Authors' contributions

JB and SR collected and analyzed the behavioral data. JB and SM drafted the manuscript. JB, TN, and SM conceived of the study, participated in its design, and edited the manuscript. All authors read and approved the final manuscript.
